# “Patient Journeys”: improving care by patient involvement

**DOI:** 10.1038/s41431-019-0555-6

**Published:** 2019-12-04

**Authors:** Matt Bolz-Johnson, Jelena Meek, Nicoline Hoogerbrugge

**Affiliations:** 1 SquareRootThinking and EURORDIS – Rare Diseases Europe, Paris, France; 2 Human Genetics, Radboud University Medical Center, Nijmegen, The Netherlands

**Keywords:** Cancer genetics, Health policy, Cancer screening, Cancer therapy

“I will not be ashamed to say ‘*I don’t know’*, nor will I fail to call in my colleagues…”. For centuries this quotation from the Hippocratic oath, has been taken by medical doctors. But what if there are no other healthcare professionals to call in, and the person with the most experience of the disease is sitting right in front of you: ‘*your patient*’.

This scenario is uncomfortably common for patients living with a rare disease when seeking out health care. They are fraught by many hurdles along their health care pathway. From diagnosis to treatment and follow-up, their healthcare pathway is defined by a fog of uncertainties, lack of effective treatments and a multitude of dead-ends. This is the prevailing situation for many because for rare diseases expertise is limited and knowledge is scarce. Currently different initiatives to involve patients in developing clinical guidelines have been taken [1], however there is no common method that successfully integrates their experience and needs of living with a rare disease into development of healthcare services.

Even though listening to the expertise of a single patient is valuable and important, this will not resolve the uncertainties most rare disease patients are currently facing. To improve care for rare diseases we must draw on all the available knowledge, both from professional experts and patients, in order to improve care for every single patient in the world.

Patient experience and satisfaction have been demonstrated to be the single most important aspect in assessing the quality of healthcare [2], and has even been shown to be a predictor of survival rates [3]. Studies have evidenced that patient involvement in the design, evaluation and designation of healthcare services, improves the relevance and quality of the services, as well as improves their ability to meet patient needs [4–6]. Essentially, to be able to involve patients, the hurdles in communication and initial preconceptions between medical doctors and their patients need to be resolved [7].

**Fig. 1:**
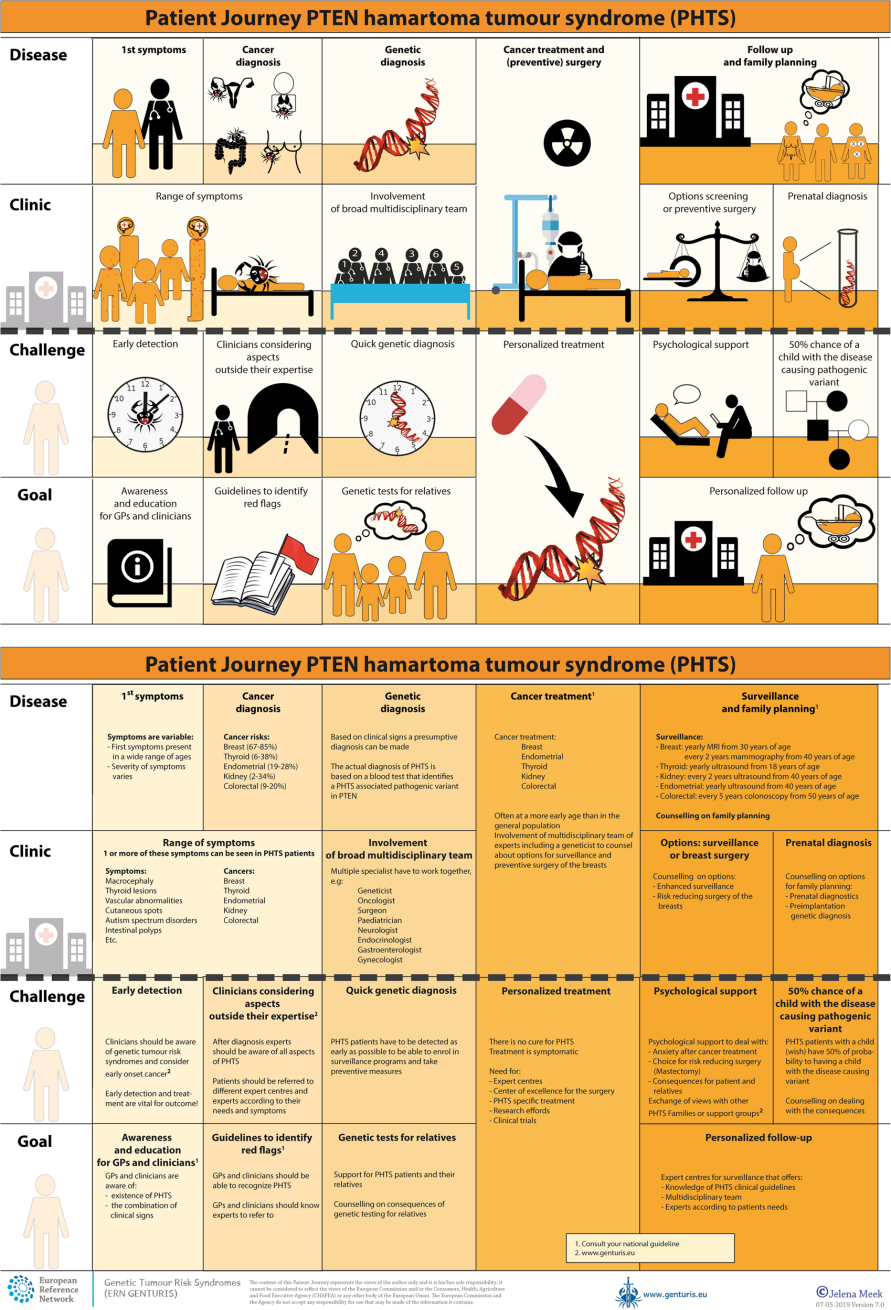
Example of a Patient Journey: PTEN Hamartoma Tumour Syndrome (also called Cowden Syndrome), including legend page (www.genturis.eu)

To tackle the current hurdles in complex or rare diseases, European Reference Networks (ERN) have been implemented since March 2017. The goal of these networks is to connect experts across Europe, harnessing their collective experience and expertise, facilitating the knowledge to travel instead of the patient. ERN GENTURIS is the Network leading on genetic tumour risk syndromes (genturis), which are inherited disorders which strongly predispose to the development of tumours [8]. They share similar challenges: delay in diagnosis, lack of cancer prevention for patients and healthy relatives, and therapeutic. To overcome the hurdles every patient faces, ERN GENTURIS (www.genturis.eu) has developed an innovative visual approach for patient input into the Network, to share their expertise and experience: “Patient Journeys” (Fig. 1).

The “Patient Journey” seeks to identify the needs that are common for all ‘*genturis syndromes*’, and those that are specific to individual syndromes. To achieve this, patient representatives completed a mapping exercise of the needs of each rare inherited syndrome they represent, across the different stages of the Patient Journey. The “Patient Journey” connects professional expert guidelines—with foreseen medical interventions, screening, treatment—with patient needs –both medical and psychological. Each “Patient Journey” is divided in several stages that are considered inherent to the specific disease. Each stage in the journey is referenced under three levels: clinical presentation, challenges and needs identified by patients, and their goal to improve care. The final Patient Journey is reviewed by both patients and professional experts. By visualizing this in a comprehensive manner, patients and their caregivers are able to discuss the individual needs of the patient, while keeping in mind the expertise of both professional and patient leads. Together they seek to achieve the same goal: improving care for every patient with a genetic tumour risk syndrome.

The Patient Journeys encourage experts to look into national guidelines. In addition, they identify a great need for evidence-based European guidelines, facilitating equal care to all rare patients. ERN GENTURIS has already developed Patient Journeys for the following rare diseases (www.genturis.eu):

- PTEN hamartoma tumour syndrome (PHTS) (Fig. 1)
- Hereditary breast and ovarian cancer (HBOC)
- Lynch syndrome
- Neurofibromatosis Type 1
- Neurofibromatosis Type 2
- Schwannomatosis

A “Patient Journey” is a personal testimony that reflects the needs of patients in two key reference documents—an accessible visual overview, supported by a detailed information matrix. The journey shows in a comprehensive way the goals that are recognized by both patients and clinical experts. Therefore, it can be used by both these parties to explain the clinical pathway: professional experts can explain to newly identified patients how the clinical pathway generally looks like, whereas their patients can identify their specific needs within these pathways. Moreover, the Patient Journeys could serve as a guide for patients who may want to write, in collaboration with local clinicians, diaries of their journeys. Subsequently, these clinical diaries can be discussed with the clinician and patient representatives. Professionals coming across medical obstacles during the patient journey can contact professional experts in the ERN GENTURIS, while patients can contact the expert patient representatives from this ERN (www.genturis.eu). Finally, the “Patient Journeys” will be valuable in sharing knowledge with the clinical community as a whole.

Our aim is that medical doctors confronted with rare diseases, by using Patient Journeys, can also rely on the knowledge of the much broader community of expert professionals and expert patients.
